# New tools to diagnose and follow FAC patients: biomarkers

**DOI:** 10.1186/1750-1172-10-S1-I16

**Published:** 2015-11-02

**Authors:** Giampaolo Merlini

**Affiliations:** 1 Amyloidosis Research and Treatment Center, University Hospital Policlinico San Matteo, Pavia, Italy

## 

In the last decade, the availability of the cardiac biomarkers N terminal pro-natriuretic peptide type-B (NT-proBNP) and troponins has radically changed the approach to diagnosis, staging, and response assessment of cardiac amyloidosis. Despite similar appearance at standard imaging, the main types of amyloidosis involving the heart are characterized by different rates of progression and different outcomes. In general, patients with ATTRm amyloidosis have lower concentrations of cardiac biomarkers and usually a better prognosis than subjects suffering from AL amyloidosis. Nevertheless, the clinical manifestations of ATTRm amyloidosis are heterogeneous. Different amyloidogenic TTR mutations give rise to different cardiac phenotypes, ranging from exclusively neuropathic diseases, through mixed phenotypes, to mutation characterized by severe cardiac dysfunction. For instance, one of the mutations associated with cardiac involvement in ATTRm amyloidosis, Ile68Leu, presents with high NT-proBNP concentrations, comparable to those observed in patients with cardiac AL amyloidosis. In our series, survival of patients with Ile68Leu ATTRm is not different from that of cardiac AL patients. Thus, it is possible that NT-proBNP represents a viable marker for early diagnosis of cardiac involvement in ATTR patients with “aggressive” mutations. This marker could be considered in the screening of carriers of these mutations. Despite the lack of systematic studies of cardiac biomarkers in ATTR amyloidosis, the results of several small published series and the analysis of the Pavia patient population, indicate that NT-proBNP and cardiac troponins have prognostic value in this disease, correlating to amyloid burden and adding to known prognostic factors. The availability of several novel therapeutic options for ATTRm, require objective means for the assessment of treatment efficacy in routine clinical practice and in clinical trials. Early results from the Pavia series indicate that NT-proBNP progression portends a poor prognosis, and cardiac biomarkers will probably find their place in the evaluation of response to therapy in ATTRm amyloidosis. Cardiac biomarkers will most likely become increasingly useful in the management of patients with ATTRm amyloidosis in the near future. However, studies of biomarkers in this disease are hindered by small numbers and disease heterogeneity. Prospective studies and international collaboration are warranted to define the role of cardiac biomarkers in the diagnosis and follow-up of cardiac ATTRm amyloidosis.

